# Medical colleges’ challenges and coping strategies in implementing accreditation standards in Pakistan

**DOI:** 10.12669/pjms.38.7.6121

**Published:** 2022

**Authors:** Fouzia Gul, Usman Mahboob, Gohar Wajid, Akhtar Sherin

**Affiliations:** 1 Fouzia Gul, MBBS, FCPS, MCPS; MHPE. Professor, Department of Gynae/Obs & HOD Department of Medical Education, Khyber Medical University, KMU Institute of Medical Sciences, Kohat, Pakistan; 2 Usman Mahboob, MBBS, MPH, FHEA (UK), DHPE (UK), Fellow FAIMER (USA) Associate Professor in Medical Education, Institute of Health Professions Education & Research. Khyber Medical University, Peshawar, Pakistan; 3 Gohar Wajid, MBBS, MSc, MPH, PhD. Health Professions Education Consultant; 4 Akhtar Sherin, MBBS, FCPS, FRCP (Glasg), MSc (Health Research) Professor, Department of Medicine, KMU Institute of Medical Sciences, Kohat, Pakistan

**Keywords:** Accreditation, Standards, Challenges, Schools, Medical, Faculty, Hospitals, Pakistan Medical Commission, Pakistan Medical & Dental Council

## Abstract

**Objectives:**

To explore the challenges faced by medical colleges and coping strategies used in implementing accreditation standards by Pakistan Medical Commission during accreditation inspection 2019.

**Methods:**

In this qualitative case study, four medical colleges and their affiliated hospitals from three cities in Khyber Pakhtunkhwa province of Pakistan were selected through purposive sampling. Data was collected through focus group discussions (FGD) through Open-ended questions, based on CIPP (context, input, process, and product) model. Each focus group comprised of Dean, the Director Department of Medical Education (DME) and the Medical Director of the hospital. Data were thematically analyzed and results were based on the CIPP model.

**Results:**

Three themes identified were *administrative challenges, accreditation challenges* and *resource challenges*. *The administrative challenges* theme was further explained under subthemes of rules and regulation challenges, documentation and record challenges, and DME-related challenges. The *accreditation-related challenges* theme was explored in-depth with subthemes of *accreditation process-related challenges, accreditation standards-related challenges* and *curriculum-related challenges*. The *resource challenges* theme was described under sub-themes of *infrastructure-related challenges, human resources* and *financial challenges*. The commonest coping strategies adopted by medical colleges were establishing DME, emergency preparatory meetings of staff, and hiring staff on an emergency basis, to overcome administrative, accreditation and resource challenges respectively. Future suggestions for improving the accreditation process in the local context were highlighted.

**Conclusion:**

Main challenges identified were administrative challenges, accreditation challenges and resource challenges. Coping strategies by the medical colleges for these challenges are highlighted. The accreditation body should harmonize the process of accreditation with medical colleges and other stakeholders.

## INTRODUCTION

The surge in global demand for health professionals and the resulting exponential increase in the number of health professions education institutions has blurred the international boundaries for medical students and physicians. The wide variations in medical training programs and the lack of robust accreditation systems have threatened the overall quality of medical education worldwide. Globally, there is increasing realization for developing global medical education standards and accreditation systems to ensure the quality of medical education. World Federation for Medical Education (WFME) published its global standards to guide countries to contextualize and develop their own standards.^1^ These standards have been adopted by many countries after necessary adaptations, including the US Education Commission for Foreign Medical Graduates.^2^

Accreditation is one of the contemporary methods for quality assurance in many fields including medical schools.^3^ However, medical education and clinical training are not uniform across the world.^4^ Considerable variations exist in accreditation systems in the form of ownership, the scope of authority, standards, process and level of enforcement.^5^ Literature identifies a range of accreditation challenges like too many standards, accreditation infrastructure, financial issues, time constraints, documentation, shortage of staff, added workload, poor survey instrument, quality of evaluators, accreditation sustainability and legal support.^6^,^7^

Pakistan Medical Commission (PMC), as the sole regulatory body carries the responsibility of accreditation for over 150 medical and dental colleges in the country. The country requires a robust accreditation system to cater the needs of such a large number of medical and dental colleges. Few important features of the system include developing and updating accreditation standards that are contextual to the needs of the country, developing policies and procedures for accreditation in consultation with key stakeholders, and ensuring that faculty and other staff are well-trained to prepare the colleges for accreditation. Research on medical accreditation is limited in Pakistan. WFME standards provide a good start to develop a robust accreditation system in the country. In 2019, Wajid et al. conducted a study to assess the relevance of WFME standards to the Pakistani context.^8^ Later on, Khan and colleagues identified the likely challenges faced by Pakistani colleges in implementing WFME standards in Pakistan.^9^

No study has been conducted in Pakistan on the experiences of key stakeholders (faculty and administration of medical colleges) about the accreditation challenges faced during the accreditation process. This study provides evidence from the first-hand experiences of key persons involved in the accreditation process, challenges faced by them while preparing the institution for accreditation and strategies adopted by them for overcoming these challenges.

## METHODS

This qualitative case study was conducted in 2020-2021, after the PMC inspection in 2019 for accreditation of medical colleges. The study was approved by the ethical committee of Khyber Medical University Peshawar, Pakistan [Reference No. DIR/KMU.EB/CF/00077 ON 06/09/2021].

### Sample Selection

Four medical colleges and their affiliated hospitals from three cities in the Khyber Pakhtunkhwa (KP) province of Pakistan were selected through a purposive, maximum variation sampling technique. The medical colleges were selected from the central, northern and southern districts of KP. Out of four medical colleges, three were public sector and one was a private medical college**.** Administrative approvals from the concerned medical colleges and written, informed consent from all participants were taken.

### Data Collection

The CIPP (*Context, Input, Process* & *Product*) model was used as a theoretical framework because of its inherent capacity for objective conclusions.^10^ All the four components of CIPP were used to analyze accreditation inspection. The *context and input* evaluation describe the challenges faced during the preparatory phase of inspection. The *process* evaluation describes challenges faced during the actual inspection and *product* evaluation is about various coping strategies and future suggestions. Completion of the accreditation process was taken as the end product. A questionnaire, based on the CIPP model was developed comprising of eight open-ended questions, covering all the components of CIPP.

Data was collected through focus group discussions (FGDs). Two FGDs were conducted face to face in medical colleges and another two, through Zoom meetings because of the Covid-19 pandemic. Each FGD lasted for 45-60 minutes. Each focus group comprised of Dean, DME director of each medical college and medical director (MD) or medical superintendent (MS) of their affiliated hospital. Proceedings of FGD were recorded and notes were taken for important points.

### Data Analysis

Audio recording of FGDs was transcribed manually and was counter-checked by an independent observer. The data was analyzed through three cycles of coding and thematic analysis was done to generate themes from these codes. The results were analyzed and compiled based on the CIPP model.

## RESULTS

Among the 12 FGD participants, 3 (25%) were females and 9 (75%) males aged from 40-50 years. The main challenges identified were grouped into three themes & nine subthemes. Theme one included administrative challenges with subthemes of rules and regulation challenges, documentation and record challenges and DME-related challenges (Table-I).

**Table I:** Administrative challenges faced during an accreditation visit.

THEME: Administrative challenges		

Subtheme	Evaluation Model	Representative quote
Rules & regulation related Challenges	Context	*“The convener of PMC new standards committee was from postgraduate training hospitals and was not very much aware about undergraduate requirements”*. (Principal-1)
	Input	*“To be very honest, to a larger extent, we were not aware of any Performa or standards. Even when the inspectors came for pilot visit, they did not have any Performa*;” **(Principal-2)**
	Process	“In hybrid composition, the coordination and goals among different stack is produced with difficulty” (MD-1)
	Product	“*Discussions with higher authorities of health department* and *medical university to come up with demands of PMC”*. (DME Director-1)
Documentation & record related challenges	Context	*“It was not an inspection but going through the record”(Principal-2)*
	Input	“*it was not accreditation rather audit of medical college”(Principal-4)*
	Process	* “If I am given the opportunity to speak the whole truth, we adopted deceptive fake and ostensible temporary measures to fill the gaps like installation of defective equipment and provision of filtered information to the PMC inspection team”(MD-2)*
	Product	*“Record was retrieved on recall basis, from old registers, purchase slips a* nd *computer data basis” (MD-2)*
DME-related challenges	Context	*“The DME was made mandatory for recognition since 2008 but was started just before visit” (DME director-1)*
	Input	*“The additional responsibility of DME was given to already over-occupied faculty” (DME director-4)*
	Process	*“PMC inspector was judging our faculty rather than standards. The PMC team came with the mind set to make us fail” (DME director-2)*
	Product	*The importance of DME was realized a* nd *faculty registered itself in various medical education certificate a* nd *masters programs (Principal-4)*

PMC: Pakistan Medical Commission; MD: Medical Director; DME: Department of Medical Education.

The second theme was accreditation challenges with sub-themes of accreditation process-related challenges, accreditation standards-related challenges and curriculum-related challenges (Table-II). The third theme was resource challenges with sub-themes of infrastructure-related challenges, human resources and financial challenges (Table-III).

**Table II:** Accreditation challenges faced during an accreditation visit.

THEME: Accreditation-related challenges		

Subtheme	Evaluation Model	Representative quote
Accreditation process-related challenges	Context	*“The accreditation process was as stressful as like thesis defence (“DME director-2)*
	Input	“*Many developments in pipeline could not be implemented be* cause *of short preparation time”* (MD-4)
	Process	*“It is very hectic for college as well as for PMC team to inspect college in one day on such elaborated proforma”* (DME director-1)
	Product	*“Single uniform policy will increase liaison between accreditation body, health and finance departments.”(Principal-4)*
Accreditation standards-related challenges	Context	“ *They just sent us the standards but how to prepare on these standards, there was no awareness” (* Principal-3)
	Input	*“The PMC should follow realistic approach according to our local conditions and such strict criteria for evaluation is very difficult to fulfil” (MD-3)*
	Process	*“The evaluation of different categories of hospitals and medical colleges on the same standards led to biased results”(Principal-4)*
	Product	*“PMC standards should be modified according to hospitals categories and for medical colleges should be adjusted according to its location/funding and facilities being given” (Principal-1)*
Curriculum-related challenges	Context	“*All of a sudden the modular system was implemented in all medical colleges. We implemented it for inspection purpose without any know-how of this system” (* DME director-4)
	Input	*“We invited medical educationist from various universities as guest speakers to give orientation & training to our faculty members*” (Principal-4)
	Process	*“Fake timetables were made. Seeing timetable only is a deceivable method*.” (DME director-1)
	Product	*“The importance of DME & integrated modular curriculum was realized.” (DME director-4)*

DME: Department of Medical Education; MD: Medical Director.

**Table III:** Resource-related Challenges faced during an accreditation visit.

THEME: Resource Challenges		

Subtheme	Evaluation Model	Representative quote
Infrastructure-related challenges	Context	*“It was for the first time that PMC inspectors were doing inspection in very detail. They practically verified the working conditions of various equipment & familiarity of staff on use of these equipment”* (MD-2)
	Input	*“The basic architecture of this hospital is not consistent with its purpose and function. Especially, at medical education site, there is no lecture hall, no demonstration room, and no library in the hospital”*. (MD-1)
	Process	*“We were in shuffling phase with split hospital having half departments in one hospital & half in other hospital. Because of this transition phase, we have been marked deficient in many equipment which we have purchased now”* (MD-4)
	Product	*“By erecting partitions, halls were converted into small rooms for small group discussion, day-care centre and offices for various departments”.* (Princiapl-1)
Human resource	Context	“*The human resources demanded by PMC is very difficult to meet in present circumstances of budget and posts allocated*.”(MD-4)
	Input	“*I was thinking very much about the difficulties which I will face in the college and the college already has problems of faculty insufficiency or deficiency’* (Principal-1)
	Process	“*Being DMS, I was responsible for hospital administration and maintenance, I am member of procurement committee, I am taking care of regional blood centre”* (MD-4)
	Product	*“New posts were created. Many vacant posts were filled”. (MD-3)*
Financial challenges	Context	*“We were very much deficient in financial resources. The release of budget from health department was lengthy procedure and needed approval from finance department i.e. KPPRA/PPRA rules.”* (MD-1)
	Input	“*We left our deficiencies as such for visit because the budget release and purchase is lengthy process. We could not expedite it for visit purpose despite of huge, allocated amount*.” (MD-4)
	Process	*“We were very much short of time for inspection so budget release for major purchases was not possible”(Principal-4)*
	Product	*“Petty cash and faculty donations helped in renovation & small purchases”* (Principal-1)

PMC: Pakistan Medical Commission; MD: Medical Director;

KPPRA: Khyber Pakhtunkhwa Public Procurement Regulatory Authority;

PPRA: Public Procurement Regulatory Authority.

## DISCUSSION

Challenges faced by medical colleges during PMC accreditation inspection 2019 were grouped under three themes: administrative, accreditation and resource challenges.

The study identified several ***administrative challenges*** at the level of PMC, medical colleges, and hospital administration during the accreditation process. These challenges mainly stem from the lack of planning, lack of coordination among different stakeholders and poor staff training for preparing and conducting accreditation. The PMC, since its inception struggled in developing and implementing its rules, regulation and policies.^11^ Administrative challenges of insufficient technical capacity, political influence and ad-hoc members in the accrediting body, and problems with rules and regulation have already been highlighted in other national and international studies.^12^,^13^ Rafi and Anwar highlighted the accreditation challenges in low and middle-income countries in a recent scoping review. These include the poor technical capacity of the accrediting body, lack of autonomy, undue political pressure, bureaucratic interference in decision making, ad-hoc members in the council and committees, bias in the selection of inspectors, incompetent inspectors, inappropriate inspection time and lack of coordination.^14^

The medical colleges and hospital administration faced various executive challenges due to variations in the administrative and financial rules and regulations of the institutions working under the Medical Teaching Institution (MTI) act and the non-MTI act. In 2015, KP Government granted financial and administrative autonomy to various medical colleges and affiliated teaching hospitals through the MTI act to improve the quality of health services and delegated administrative powers and responsibilities to an independent policy board.^15^ The health and finance departments were neither involved in developing standards nor taken on board during accreditation inspection. All medical colleges and affiliated hospitals were assessed with the same yardstick, although they were being governed under different rules. This ‘one-size-fits-all’ approach led to several administrative and financial challenges. Braithwaite et al. (2012) mentioned similar administrative and expenditure challenges in low- and middle-income countries as the main obstacle to successful and sustainable accreditation.^16^ Accreditation is a collective process with a distinct role played by key stakeholders in achieving the desired results. All stakeholders must be taken in confidence and play an effective role in making accreditation successful from its planning to final execution. Failure to effectively involve stakeholders in accreditation planning and execution may threaten the effectiveness of accreditation. It may become merely a tick box exercise.

Though the department of medical education (DME) was declared the accreditation of institutions in 2008, the accrediting body did not provide the regulations and policy guidelines for establishing DMEs.^17^ The role of DME remained ambiguous and inactive in medical colleges due to the lack of guidelines, absence of full-time DME directors, inadequate infrastructure and human and financial resources for DME.^18^ Along with inadequate infrastructure for DME departments, the lack of training of DME staff poses a major threat to the efficient and effective functioning of the departments. Although health professions education programmes have taken their roots in Pakistan over the past one decade, effective training of DME staff, especially for planning and conducting accreditation at institutional level still remains questionable.

One of the biggest challenges faced by almost every medical college was a poor documentation system. Short preparatory time, variation in rules and regulations and non-familiarity with new standards resulted in an increased workload on faculty and hospital administration. Similar accreditation challenges of increased workload, faculty resistance and too many standards were reported in a study from Iran.^19^

Participants of our study mentioned several coping strategies to get through the accreditation process successfully. Most strategies were mere box-ticking exercises to maximize accreditation scores, rather than addressing the gaps in the quality of education in a more transformative and sustainable manner. Few strategies included establishing DME (albeit on papers), manipulation of records and creating new documents on an emergency basis.

Some of the challenges faced during the inspection were solely related to the ***accreditation body and the planning and process of accreditation***. Proficient and skillful inspectors are vital to conducting the accreditation survey effectively. Inspectors must be trained well on the nature and measurement of standards, and their role during the accreditation process. Unfortunately, the PMC overlooked both the selection and training of its inspectors, which resulted in an inconsistent inspection process. One participant reported that the inspectors finished the whole inspection process in a college in single day only (typically eight hours). Such threat to the validity of the accreditation process has been reported in other studies from Pakistan and Iran.^9^,^12^ In one of the colleges, the inspection was carried out during the month of July, without considering summer vacations and hot weather. Vali (2020) also reported similar incompatibility in scheduling accreditation in Iran.^19^ The successful implementation of accreditation standards can be ensured when the standards are contextualized to each country’s needs and accepted by health professionals.^20^ Accreditation can serve as a strong driving force to upgrade educational programs, including the curriculum.^21^ The quality of medical education in Pakistan is compromised due to a lack of curricula based on international standards.^9^,^11^ Non-alignment of educational and medical accreditation policies results in the wastage of resources and the purpose of accreditation cannot be achieved.^22^ The independent nature of accreditation department, availability of highly trained staff, well developed standards of education that are thoroughly understood and implemented by the institutions and a major shift in the perception of accreditation as a ‘policing exercise’ to a ‘quality improvement’ strategy is likely to help the institutions reap the real rewards of accreditation.

Coping strategies included calling emergency staff meetings to distribute responsibilities to prepare the college for accreditation, staff orientation on accreditation standards and identifying the major deficiencies that could drastically pull the score down. It was evident that there was no concerted quality improvement effort behind the accreditation process. The curricular issues were addressed by medical colleges through the capacity building of faculty members. Developing new teaching strategies, incorporation of study guides, adjustment of subject hours in timetable, improved internal assessment and relevant documentation were the main reforms in the curriculum.

Medical colleges and hospital administration faced various ***resource challenges:*** human resources, infrastructure, and financial difficulties. PMC has set standards for human resources in medical colleges and affiliated hospitals. These resources include a range of faculty, office personnel and other support staff. Medical colleges generally faced the challenges of fulfilling human resource requirements as per PMC standards. Studies conducted in Pakistan^11^ and Bangladesh^23^ highlighted similar human resource shortage challenges. Participants mentioned that the buildings of medical colleges and their affiliated hospitals, especially in the periphery were not purpose-built. Studies from Bangladesh^23^ and India^24^ pointed out similar challenges of inadequate infrastructure, insufficient staff, and weak logistics management in accreditation implementation. Implementing a national accreditation program requires considerable financial resources.^25^ Participants complained that the inspection was carried out in urgency, ignoring the financial constraints of medical colleges and their affiliated hospitals. The release of finances to fulfil financial commitments required coordination between Finance and Health Departments, which was simply missing. This led to the lack of financial availability to comply with PMC accreditation standards. Institutions had to hire staff on an emergency basis to fill staff deficiencies. The finance department was requested to release funds in an emergency to buy necessary equipment on urgent basis to fulfil standards requirements. At times, accreditation of an institution might be a time consuming and resource intensive exercise. A robust accreditation system is still far from taking its roots in Pakistan. Communication gaps and the lack of coordination among stakeholders could pose serious threats to the successful implementation of the system. These gaps must be addressed by the leadership.

Participants gave several suggestions to improve the accreditation process. These related to the composition of the accreditation team, inspection schedule, inspectors training, liaison between health and finance departments, better coordination between the colleges and PMC and post-inspection analysis. The inspection of an institution for accreditation purposes is a resource intensive process. PMC may consider introducing a pre-inspection process to guide the institutions conduct their self-assessment for readiness for inspection. Effective staff training at all levels is the key to successful accreditation.

### Limitations

This study was conducted in just four medical colleges of KP province, so it cannot be generalized to all medical colleges of Pakistan because of different administrative and financial rules and regulations in other provinces. The accreditation challenges could be explored more holistically by interviewing the heads of the inspection committees and conveners of the accreditation team. Medical colleges in the public and private sectors may also need to be explored further as the challenges may be different for the two sectors.

## CONCLUSION

The main challenges faced by medical colleges during accreditation inspection were related to administration, accreditation body and resources. The main administrative challenges faced were related to records and documentation followed by highly variable regulations among public and private sector medical colleges and hospitals. The significant resource-related challenges were meeting human resource requirements, poor infrastructure and financial constraints. Untrained inspectors, poor inspection scheduling and lack of uniform inspection standards were the accrediting body related challenges.

## RECOMMENDATIONS

The study provides evidence not only about the challenges during the accreditation process but also shows how institutions view accreditation as a policing and audit process. Quality assurance is the essence of any accreditation process. PMC needs to develop a robust accreditation system in consultation with the stakeholders. An accreditation department at PMC that is equipped with technically qualified staff and leadership support to develop and implement an accreditation system in over 150 medical and dental colleges of the country is perhaps the biggest challenge faced by the Commission and the colleges. Effective stakeholder coordination and staff training at all levels may significantly improve the accreditation system in Pakistan.

### Author’s Contribution:

**FG:** Conceptualization, data collection, data analysis. Writing the manuscript and final editing of the manuscript. Approval of the final manuscript

**UM:** Conceptualization, developed methodology, analyzed data, critical revision manuscripts and final editing of the manuscript. Approval of the final manuscript

**GW:** Conceptualization, developed methodology, critical revision of all drafts and final editing of the manuscript. Approval of the final manuscript

**AS:** Data collection and analysis. Manuscript writing. Approval of the final manuscript

All authors agree to be accountable for all aspects of the work in ensuring that questions related to the accuracy or integrity of any part of the work are appropriately investigated and resolved.
